# Radio-Frequency Electromagnetic Field Exposure of Western Honey Bees

**DOI:** 10.1038/s41598-019-56948-0

**Published:** 2020-01-16

**Authors:** Arno Thielens, Mark K. Greco, Leen Verloock, Luc Martens, Wout Joseph

**Affiliations:** 1 Ghent University - imec, Department of Information Technology, Ghent, B-9052 Belgium; 2 University of California Berkeley, Berkeley Wireless Research Center, Department of Electrical Engineering and Computer Sciences, Berkeley, CA 94704 USA; 3 Charles Sturt University, Medical Imaging, SDHS, Faculty of Science, Wagga Wagga, NSW 2678 Australia

**Keywords:** Entomology, Environmental sciences, Electrical and electronic engineering

## Abstract

Radio-frequency electromagnetic fields (RF-EMFs) can be absorbed in all living organisms, including Western Honey Bees (*Apis Mellifera*). This is an ecologically and economically important global insect species that is continuously exposed to environmental RF-EMFs. This exposure is studied numerically and experimentally in this manuscript. To this aim, numerical simulations using honey bee models, obtained using micro-CT scanning, were implemented to determine RF absorbed power as a function of frequency in the 0.6 to 120 GHz range. Five different models of honey bees were obtained and simulated: two workers, a drone, a larva, and a queen. The simulations were combined with *in-situ* measurements of environmental RF-EMF exposure near beehives in Belgium in order to estimate realistic exposure and absorbed power values for honey bees. Our analysis shows that a relatively small shift of 10% of environmental incident power density from frequencies below 3 GHz to higher frequencies will lead to a relative increase in absorbed power of a factor higher than 3.

## Introduction

Wireless communication is a widespread and growing technology. Most of the wireless networks and personal devices operate using Radio-Frequency (RF) electromagnetic fields (EMFs). The current networks rely on frequencies between 0.1 GHz and 6 GHz^1^. These EMFs can be absorbed in dielectric media and can cause dielectric heating^2^. This dielectric heating can occur in any living organism, including insects.

Absorption of RF EMFs in insects has been studied previously. Wang *et al*.^3^ studied absorption of RF EMFs in mashed codling moth larvae at 27 MHz and 915 MHz. Shrestha *et al*.^4^ studied dielectric heating of Cryptolestes ferrungineus S. in different stages (eggs, larvae, pupae, and adults) at 27 MHz. Shayesteh *et al*.^5^ exposed Tribolium confusum and Plodia interpunctella to RF EMFs at 2450 MHz^6–8^. are reviews of RF heating of insects. Dielectric porperties of insects are measured by Nelson *et al*.^9^ from 0.2 to 20 GHz through the determination of loss of RF EMF power in insect samples (rice weevil, red flour beetle, saw-toothed grain beetle, and lesser grain borer). Absorption of RF EMFs was studied by Halverson *et al*.^10^ in insects between 10–50 GHz. Thielens *et al*.^11^ used numerical simulations to study absorption of RF EMFs from 2–120 GHz in four insect models. The main conclusions from the aforementioned studies are that (i) RF EMFs can be absorbed and can cause dielectric heating in insects and (ii) this absorption of RF-EMFs is frequency dependent. This frequency dependency is important since 5th generation (5 G) networks are expected to partially operate at higher frequencies (up to 300 GHz)^12,13^. This shift might induce a change in RF EMF absorption for insects^11^.

Western Honey Bees (*Apis Mellifera*) are particularly important insects because of the environmental and economical importance of this species. Therefore, previous studies have focused on the potential effects of EMF exposure of Western Honey Bees. Low-frequency EM properties and exposure of honeybees was studied in^14^. The influence of Low-frequency magnetic fields on honey bee orientation has been studied in^15^. There have also been some studies on effects of RF EMF on honey bees. Potential effects of RF EMF exposure on reproduction of honey bee queens were investigated in^16^. Behavioral effects potentially caused by exposure to RF EMFs in honey bees have been investigated in^17–19^. A disadvantage is that these studies are lacking a quantification of the amount of power that is absorbed in the studied honey bees, so called RF dosimetry^20^. On the other hand, this absorption has been determined for a single honey bee worker in^11^. However, Thielens *et al*.^11^ do not provide any coupling of this absorption to a real RF-EMF exposure situation and only study a single honey bee, which provides no information on the evolution of such absorption as a honey bee goes through different developmental stages. Nor is it clear whether this RF absorption is realistic for other castes, such as drones or queens, in a bee colony.

Therefore, the aims of this study were to numerically evaluate RF-EMF absorption in western honey bees and validate the frequency dependency of this absorption during various developmental stages and experimentally quantify real-life exposure of bees. To this aim, numerical simulations were executed to determine the absorption of RF-EMFs in five different honey bee models: a larva, a queen, two workers, and one drone, obtained using micro-CT imaging. These simulations were implemented as a function of frequency in a broad band, 0.6 GHz up to 120 GHz, that can be used to model both current and future telecommunication frequencies. In parallel, RF-EMF exposure measurements were executed near five bee hives in Belgium, in order to quantify the real exposure of such honey bees. Finally, these measured values were used to rescale the numerical simulations in order to quantify real honey bee absorption and assess a potential change in absorption in case a shift in operation frequencies in future telecommunication networks would occur.

## Methods

### Studied honey bees, imaging technique, and model development

**Figure 1:**
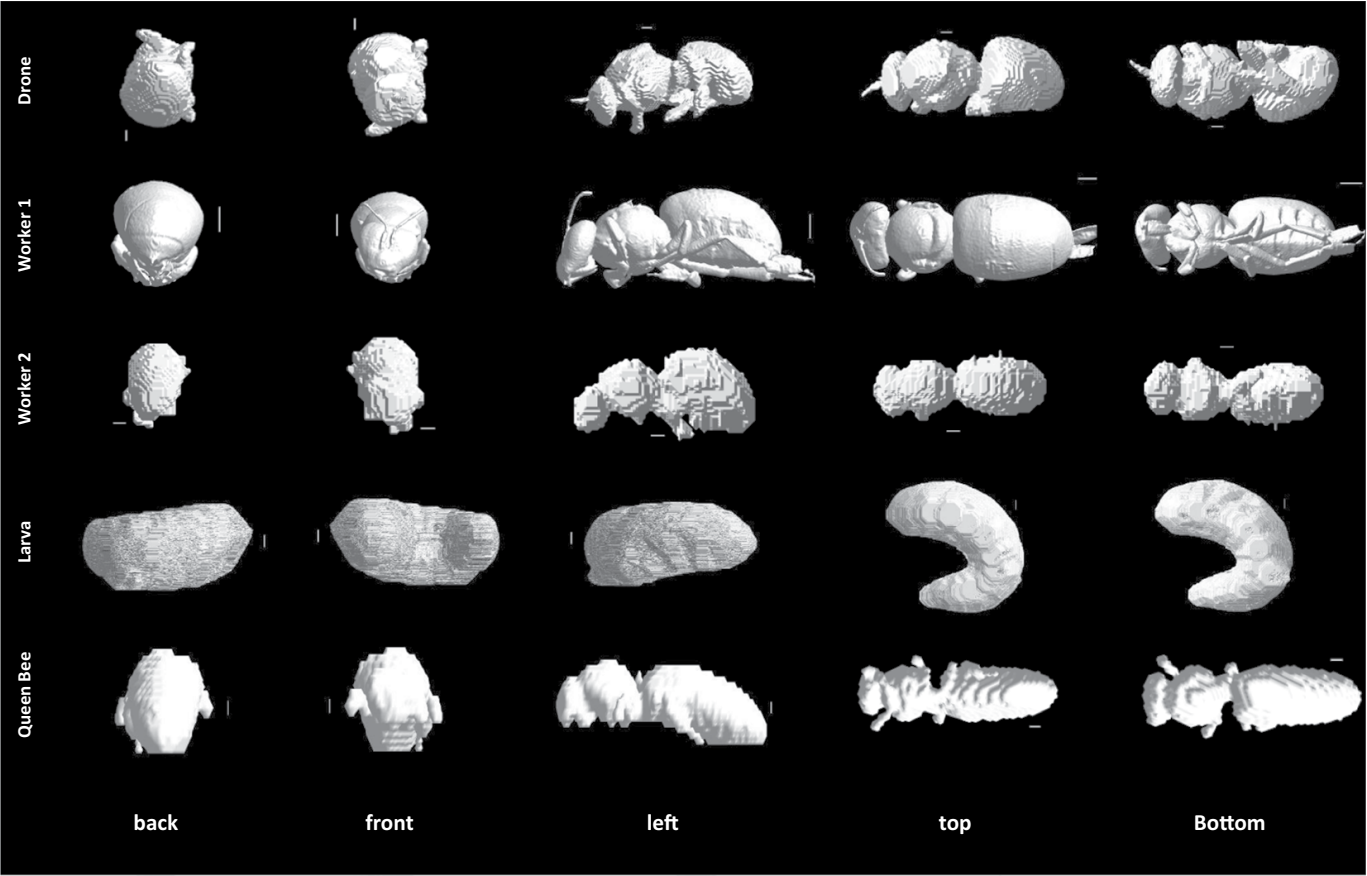
Studied Honey Bee Models, from top to bottom: Male Drone, Worker Bee 1, Worker Bee 2, Worker Larva and Queen Bee. Columns show different perspectives: back, front, left, top, and bottom view, respectively. The white lines show a 1 mm scale for reference.

Images of the studied insects are shown in Fig. 1. All studied insects are western honey bees (*Apis mellifera*), which is the most commonly used honey bee worldwide. Honey bees within a colony are subdivided into different castes. An active viable honeybee colony contains only one queen bee who spends most of her time laying 2,000 to 3,000 eggs per day. The queen is the only reproductive female within the colony and her health is vitally important to the survival of her colony. Damage to her ovaries has the potential to effect the function and survival of her progeny. A queen typically lives between approximately three and five years. From early spring time to mid-summer the queen lays unfertilized “haploid” eggs which develop into drone bees. All drones are males. Their specific role is to mate with a virgin queen so that she can initiate the propagation of a new colony. During this mating season, there are approximately 3,000 to 5,000 drones within any given colony. Drones typically live between one to two months.

A healthy honey bee colony can contain approximately 50,000 individuals. Most of these are sterile, female, worker bees. Worker bees perform all the tasks within a colony to keep it full of provisions and free from disease. This involves feeding and nursing larvae, foraging for nectar and pollen, storing nectar and pollen, guarding the entrance, tending to the hygiene of the queen-workers-drones and maintaining a clean hive environment. Workers live for three to four weeks during the active seasons (spring-summer-autumn) and approximately three months during the colder inactive season (winter). There are approximately 3,000 (winter) to 10,000 (summer) larvae present at any given time.

We chose representatives from all three castes within a honeybee colony, one queen bee, two worker bees, one drone bee and one worker larva. All honey bees were scanned at the Western Sydney University National Imaging Facility (Sydney, Australia) using a bench-top MicroCT scanner (Quantum GX MicroCT Imaging System, PerkinElmer, Hopkinton, MA, USA). The parameters used during this scanning depended on the scanned bee. Such scans are made using different projections, at different time intervals on the scanners settings. The rotation between projections also depends on the scanner’s settings and the studied honey bee (see below for full description).

#### Worker 1

The insect named ‘Worker 1’ is the same bee studied in^11^, which had a full body length of approximately 11.0 mm long, is 5.0 mm wide, and had a mass of approximately 900 mg. During the scanning of Worker 1, the Micro-CT scanner was operated using the following parameters: 50 kVp, 80 mA, and a 2048 × 2048 pixels image matrix. This resulted in scans with a 20 *μ*m isotropic voxel size. Each projection had a scanning time of 3.0 s, with 3.0 s rotation time in between projections. The total scan time for Worker 1 was approximately 18 min.

#### Worker 2

The second honey bee worker (Worker 2) has a full body length of 13 mm with cross sectional dimensions of 6.8 mm and 5.4 mm and a mass of approximately 900 mg. For Worker 2, the scanner was operated using the following parameters: 40 kVp, 70 mA, and a 2048 × 2048 pixels image matrix. The isotropic voxel size was 100 *μ*m. Each projection had a scanning time of 1.5 s. There was a 3.0 s rotation time in between each projection. The total scan time for the whole bee was approximately 10 min.

#### Larva

Larvae of this age (three weeks) are typically approximately 16 mm long with an approximate mass of 900 mg. The scanned larva was curled up, which made estimating its full body dimensions difficult, but the sample fitted within a 14 × 7 × 15 *mm*^3^ box. This scanning of the larva was done using the following parameters: 50 kVp, 80 mA, and a 2048 × 2048 pixels image matrix. This resulted in scans with a 20 *μ*m isotropic voxel size. Each projection had a scanning time of 3.0 s. and with a 3.0 s rotation time this resulted in a total scan time for the larva of 18 min.

#### Male drone

The drone has a full body length of 18 mm with cross sectional dimensions of 7.2 mm and 9.4 mm and an approximate mass of 1 g. During the scanning of the drone, the Micro-CT scanner was operated using the following parameters: 40 kVp, 70 mA, and a 2048 × 2048 pixels image matrix. The isotropic voxel size was 100 *μ*m. Each projection had a scanning time of 1.5 s. The full scan took 180 projections and there was a 3.0 s rotation time in between each projection. The total scan time for the whole bee was approximately 10 min.

#### Queen bee

The QB has a full body length of 19 mm and cross sectional dimensions of 7.5 times 7.1 *mm*^2^ and an approximate mass of 1100 mg. The queen was scanned was using the following parameters: 40 kVp, 70 mA, and a 2048 × 2048 pixels image matrix. The isotropic voxel size was 250 *μ*m. Each projection had a scanning time of 1.5 s. There was a 1.5 s rotation time in between each projection. The total scan time for the queen bee was approximately 10 min.

#### Development of 3D models

The software running on the Quantum GX, bench-top MicroCT scanner was used for all honey bees to reconstruct the 180 projection images. Those were then converted into a 2D rendered image stack of 512, 16 bit bitmap images. Finally, the BeeView volume rendering software (DISECT Systems Ltd, Suffolk, UK) was used to acquire Bee volume data from the image stack. All 3D models of the insects were created using the software TomoMask (www.tomomask.com). We used the same approach as in^11^. The image stack for each honey bee was imported into TomoMask, which also required the pixel and slice spacing. The software generated a 3D model using a marching cubes algorithm^21^. This model was then exported as an STL (STereo Lithography)^22^ file. This is a commonly used format to describe surface geometry. The models were also smoothed using the Taubin *λ*/*μ* smoothing scheme^23^ implemented in MeshLab^24^. The dimensions of the models and mesh integrity were checked (and corrected if necessary) before simulations using Netfabb (Autodesk, San Rafael, CA, USA).

### Numerical simulations and RF EMF exposure conditions

**Figure 2:**
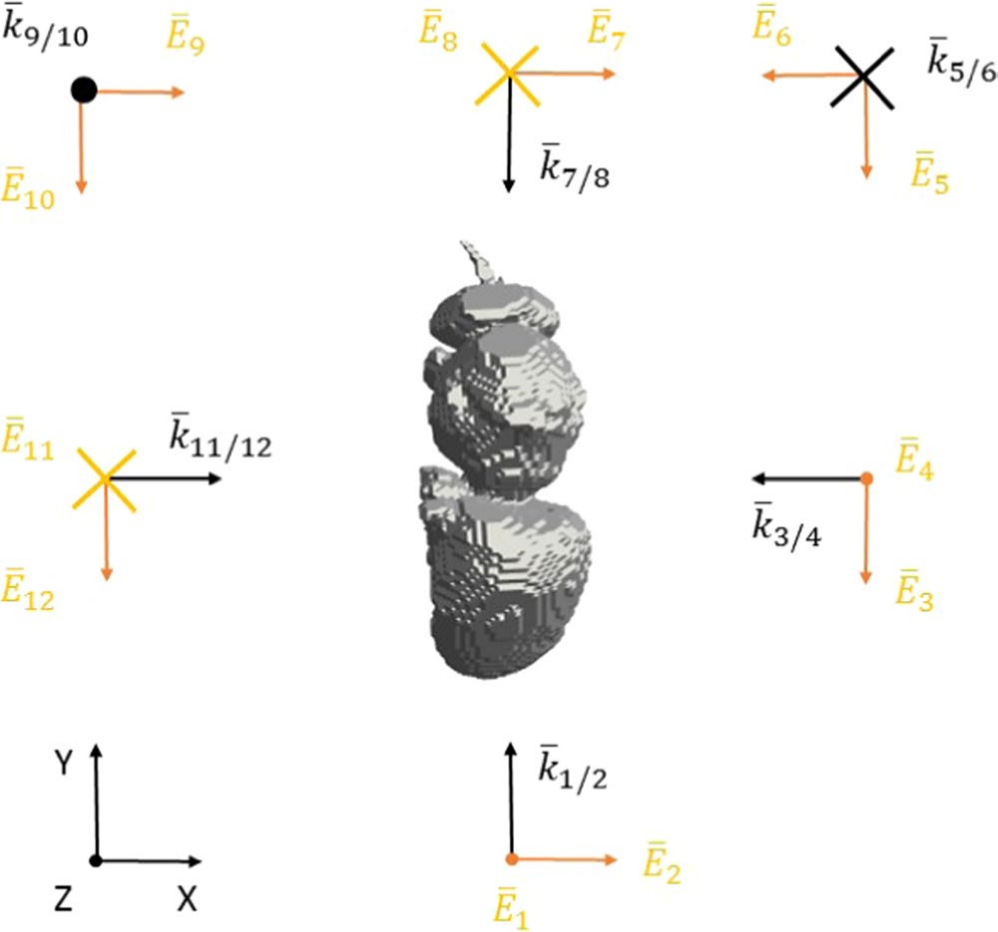
Configuration of the RF-EMF plane-wave simulations. Twelve potential RF plane waves incident from six directions are incident on the insect (honey bee drone shown here in grey, top view). Orange arrows indicate the electric field ${\overline{E}}_{i}$ polarizations, while the black arrows indicate the direction of propagation with wave vector ${\overline{k}}_{i/j}$ of the plane waves. *i* and *j* indicate the simulations’ configuration number, from 1 to 12.

Electromagnetic, numerical simulations were executed to estimate electromagnetic fields in and around the honey bees under far-field exposure. Far-field exposure is in this manuscript defined as RF-EMF sources being more than 2*D*^2^/*λ* away from the insects, with *D* the largest dimension of the RF source and *λ* the wavelength of the RF-EMFs. This is often referred to as the Fraunhofer far-field limit^25^. In general, far-field RF-EMF sources can be located in any direction from the honey bees. Therefore, different approaches exist to model such far-field exposure to RF-EMFs: a stochastic method where far-field exposure is decomposed in sets of plane waves according to certain statistics is used in^26,27^, while a more limited set of plane-wave exposures coming from six predefined directions along the main axis of the exposed subject or animal are considered in^11,28^. In this study, we have chosen to work with the latter method. We have modeled exposure of the studied honey bees by a set of 12 incident plane waves traveling along six directions defined by a Cartesian coordinate system, see Fig. 2. For each direction, two orthogonal incident electric field polarizations were chosen, since any other free-space E-field polarization can be obtained using a linear combination of both. All incident plane waves have a root-mean squared electric field strength of 1 V/m. This value is chosen to facilitate renormalization to any potential value of incident field strenght.

Numerical simulations were executed using the Finite-Difference Time-Domain (FDTD) method implemented in Sim4life (ZMT, Zurich, Switzerland). This is a common technique used to determine RF-EMF in and near homogeneous and heterogeneous dielectric objects^11,26,28^, such as the honey bees studied in this paper. In this method, the simulation domain is divided in cubes using a three-dimensional rectilinear grid. Depending on the wavelength, feature sizes of the objects in the simulations, and the desired spatial accuracy, a different spatial step is used to discretize the simulation. The FDTD algorithm requires a grid step smaller than one tenth of the smallest wavelength in the simulation domain in order to return stable solutions^29^. Since this is a time-domain technique, it requires a predefined simulation time in order to reach a steady-state solution, which will again depend on the chosen spatial resolution, the wavelength, and the size of the simulation domain.

We executed numerical simulations at nine harmonic frequencies from 0.6–120 GHz (sinusoidal waves at a single frequency). The lower and upper frequency limits were chosen because they correspond to the current limits in terms of simulation size and length that can realistically be supported by our simulation hardware. The simulated frequencies are listed in Table 1 alongside the chosen grid steps in the simulation domain and the number of periods used for every simulation. These settings were the same for each of the five studied honey bee models. The studied insects have certain dielectric properties, quantified using the relative permittivity (*ε*_*r*_) and conductivity (*σ*). We did not measure the dielectric properties of the studied insects. Instead, we assigned dielectric parameters obtained from^11^. The value at 1 GHz is obtained using the same literature database and interpolation presented in^11^. Table 1 lists these properties. All insects were modeled as homogeneous objects. These configurations resulted in 12 (plane waves) × 9 (frequencies) × 5 (honey bees) = 540 simulation results.

**Table 1:** Simulations Settings and Dielectric Properties of the Honey Bees.

	0.6 GHz	1.2 GHz	2 GHz	3 GHz	6 GHz	12 GHz	24 GHz	60 GHz	120 GHz
Maximal grid step (mm)									
Larva	0.2	0.2	0.2	0.2	0.2	0.1	0.1	0.1	0.1
Others	0.1	0.1	0.05	0.05	0.05	0.05	0.05	0.05	0.05
Simulated Periods									
Worker Bee 1	20	30	60	30	30	30	30	40	40
Others	10	20	20	30	30	30	30	30	30
${\varepsilon }_{r}$	45.6	44.2	39.9	38.8	38.0	28.6	14.9	7.018	5.46
$\sigma$ (S/m)	0.688	0.924	1.35	2.05	5.05	12.0	21.1	27.9	29.2

After each simulation, the internal electric field in the insect model was extracted and used to calculate the total absorbed RF-EMF power (*P*_*abs*_) in the honey bee. *P*_*abs*_ is calculated as the integrated product of the conductivity and the squared internal electric field strenght (${\overline{E}}_{{int}}$) over the total volume (*V*) of the insect:

1 $${P}_{abs}={\int }_{V}\,\sigma \times |{\overline{E}}_{{int}}{|}^{2}.dV$$

We report *P*_*abs*_ rather than specific absorption rate (*SAR*) values since we did not measure the mass and density of all the simulated honey bees. *P*_*abs*_ is an important quantity since dielectric heating of an insect is proportional to absorbed RF-EMF power^2^.

In order to validate our simulations we tested the influence of four simulation settings on the RF-EMF *P*_*abs*_: grid step size, dielectric parameters, angle of incidence, and number of simulated periods. The influence of the grid step is expected to be the most significant at the highest simulated frequency (120 GHz), since the chosen maximal grid step of 0.05 mm is closest to the smallest wavelength in the simulation domain at that frequency in the tissue (0.05 *mm* = 0.045 *λ*). Therefore the maximal grid step was set to 25 *μm* for exposure configuration number 2 in Fig. 2 for both the Larva and Worker 2 phantoms. In^11^, it was demonstrated that the maximal uncertainty on the dielectric parameters occurs between 2 and 3 GHz, with maximal relative deviations of 40%. In order to test the dependency of our simulation results on the chosen dielectric parameters, we executed four additional FDTD simulations in exposure configuration number 2 shown in Fig. 2 using the Worker 2 phantom. In these simulations the dielectric parameters (*ε*,*σ*) were changed to: (1.5.*ε*, 1.5.*σ*), (0.5.*ε*, 1.5.*σ*), (1.5.*ε*, 0.5.*σ*), and (0.5.*ε*, 0.5.*σ*), respectively, allowing for a potential 50% deviation on the dielectric parameters, which should be larger than the uncertainty on the chosen dielectric parameters. We chose to model RF-EMF exposure of the studied honey bees using plane waves incident from 6 directions. However, it is uncertain whether this set of plane waves provides a complete overview of the full range in *P*_*abs*_ as function of the angle of incidence. In order to validate our exposure set up, we have executed 20 additional FDTD simulations at 6 GHz using the Worker 2 phantom, where the elevation, azimuth, and polarization angles were generated according to uniform distributions between [0, *π*], [0, 2*π*], and [0, 2*π*], respectively. The settings of these FDTD simulations were the same as those shown in Table 1. Finally, the number of simulated periods was tested at 120 GHz for the Worker 2 phantom in exposure configuration number 2 shown in Fig. 2 by increasing the number of simulated periods to 120 instead of 30, see Table 1. After each of these validation simulations, the *P*_*abs*_ was extracted and compared to the one obtained in the original simulation set.

### RF-EMF field measurements

**Figure 3:**
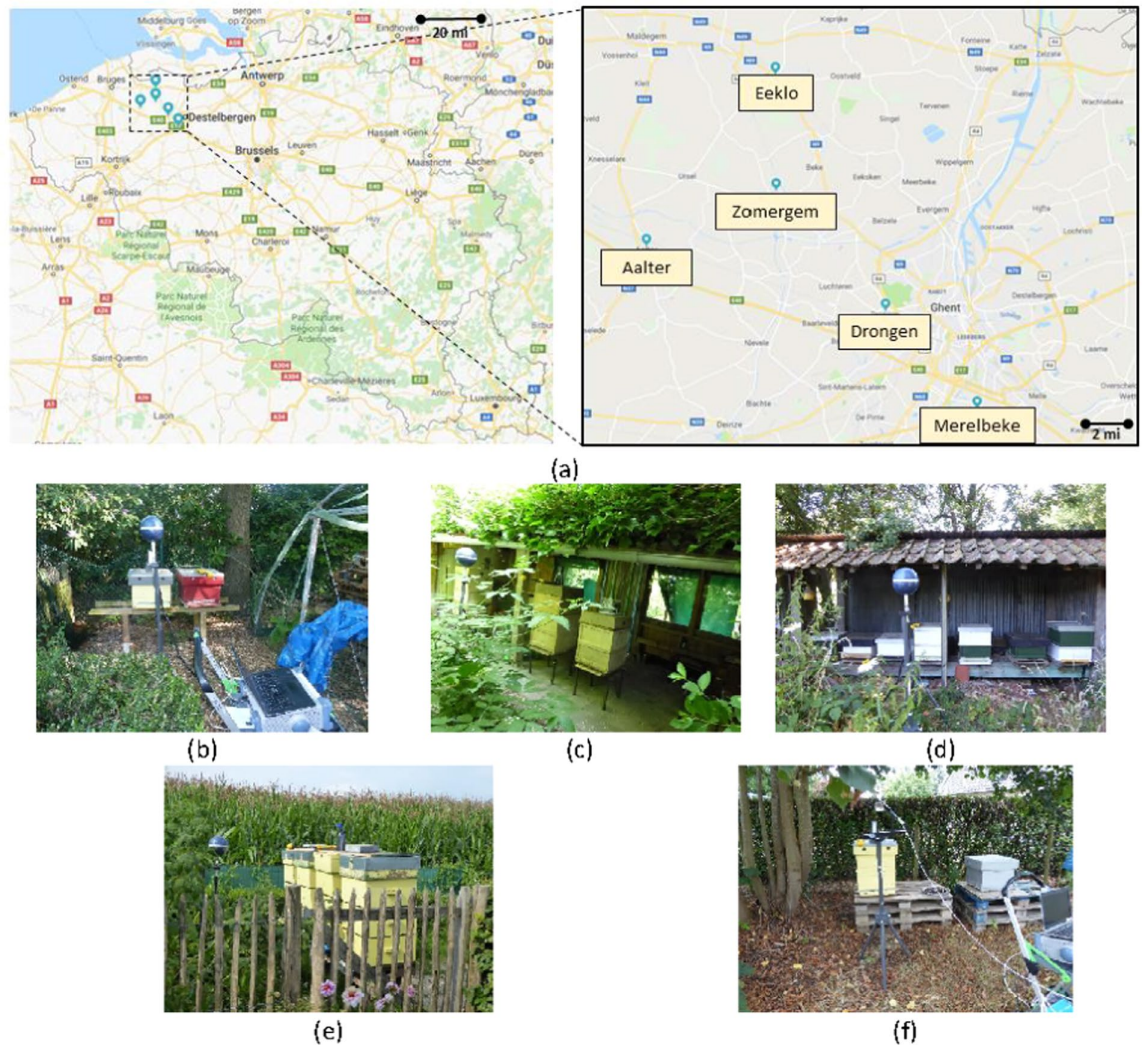
Five measurement locations near bee hives in Belgium: (**a**) Overview of the measrurement locations (source: https://www.google.com/maps, Google Maps, Google, Alphabet inc., Mountain View, CA, USA) Map data: Google, GeoBasis-DE/BKG (**b**) Aalter, (**c**) Merelbeke, (**d**) Eeklo, (**e**) Zomergem, and (**f**) Drongen.

In order to quantify current RF-EMF exposure of honey bees in real exposure scenarios, we executed RF-EMF exposure measurements at five sets of bee hives in Belgium at: Aalter, Merelbeke, Eeklo, Zomergem, and Drongen, see Fig. 3(a). At each measurement site, three different measurements were executed in order to quantify RF-EMF exposure.

First, a spectrum analyzer of the type FSL6 (R&S Belgium, Excelsiorlaan 31 1930 Zaventem Belgium) connected to a triaxial isotropic antenna was used to perform a broad-band RF overview measurement from 80 MHz to 6 GHz. These measurements were executed in two steps: first spectral overview measurements were executed from 0.08–3 GHz using a tri-axial antenna TS-EMF (Rhode and Schwartz, dynamic range of 1 mV/m–100 V/m for the frequency range of 80 MHz–3 GHz), followed by measurements from 3–6 GHz using a Clampco AT6000 antenna. At one out of five measurement sites, Drongen, a conical dipole antenna PCD 8250 (Seibersdorf Laboratories, Seibersdorf, Austria) was used for the 80 MHz - 3 GHz measurements. This antenna was rotated to obtain three orthogonal polarizations of the electric field. During these overview measurements, the spectrum analyzer measured in maximum-hold modus during 17 and 9 minutes in the lower and higher frequency bands, respectively. The antennas were supported by a plastic tripod and were placed at 1 m in front of the bee hive at a height of 1.5 m from the ground level. Figure 3 shows the studied bee hives and the measurement set up in the field. The 1.5 m height is a typical height at which such EM field measurements^30^. Additionally, this height is mentioned in the ECC(02)04 standard^31^. The purpose of these measurements was to get an overview of which frequency bands were in use at the respective sites. These frequency bands were then investigated further in the second measurements.

Second, the same spectrum analyzer was connected to the tri-axial antenna TS-EMF which was again supported by the same tripod at a height of 1.5 m. The tripod was placed at two distances of 1 and 2 m from the central bee hive. The spectrum analyzer performed root-mean square electric field strength (*E*_*RMS*_) measurements over a measurement period of 6 minutes^2^ in each of the telecommunication frequency bands identified using the first measurement. Each of the three electric field components (*E*_*x*_, *E*_*y*_, *E*_*z*_) were measured individually. *E*_*RMS*_ was then obtained as the square root of the sum of squares of the individual components.

2 $${E}_{RMS}=\sqrt{{E}_{x}^{2}+{E}_{y}^{2}+{E}_{z}^{2}}$$

The spectrum analyzer measurements in terms of received power on the antenna were then recalculated using the known antenna factor of the tri-axial antenna to incident root-mean-squared electric field strength. The *E*_*RMS,i*_ values in each frequency band (*i*) were then summed quadratically and the square root of that sum is listed as the total instantaneous electric field strength (*E*_*RMS*,*tot*_).

3 $${E}_{RMS,tot}=\sqrt{\sum _{i}\,{E}_{RMS,i}^{2}}$$

The measurement procedure and measurement settings for these RF-EMF exposure measurements are presented in^32^. The expanded measurement uncertainty (95% confidence interval) for electric field strength measurements using this set up is ±3 *dB*^30^.This measurement setup enables the most accurate assessment of *in situ* exposure from various RF-EMF sources^30^.

Third, a broadband exposure measurement was executed using a Narda NBM-550 probe (Narda, Hauppauge,NY, USA) connected to an EF 0691 broad-band probe (Narda, Hauppauge, NY, USA) which has a frequency span from 100 kHz to 6 GHz, thus including so-called intermediate frequencies (IF). These IF fields are not considered in our numerical simulations. However, we measured those to provide a complete overview of the exposure to electromagnetic field below 6 GHz. The NMB probe was placed on top of the central bee hive and was left there during both RF measurements. The device measured and registered root-mean-squared electric field strengths with a period of 1 s. From those time series of measurements, we obtained the time average and the maximal value.

The researchers that executed the RF-EMF field measurements did not use personal devices during the measurements. All wireless devices brought to the measurement site by the researchers were operated in flight mode, i.e. any wireless transmissions by those devices were not allowed.

### Estimation of realistic RF-EMF absorbed power in honey bees

Realistic *P*_*abs*_ absorbed in honey bees can be obtained by rescaling the simulated *P*_*abs*_ values using the measured incident field strengths. Therefore, we linearly averaged the total *E*_*RMS*_ values measured near the five bee hives at two different positions to obtain an average *E*_*RMS,avg*_ value. In order to estimate exposure of honey bees in current wireless networks, we averaged the *P*_*abs*_ values using:

4 $${P}_{abs,av}(f < 3\,GHz)=\frac{1}{4}\,\mathop{\sum }\limits_{i=1}^{4}\,{P}_{abs}({f}_{i})$$

with *f*_*i*_ = 0.6, 1.2, 2, 3 *GHz*. We only considered *P*_*abs*_ values < 3 GHz, since our measurements will show that there are only incident RF-EMFs below 3 GHz in the current environment of honey bees in Belgium. This value is then rescaled using:

5 $${P}_{abs,real}(f < 3\,GHz)=\frac{{E}_{RMS,avg}^{2}}{1\,{V}^{2}/{m}^{2}}\times {P}_{abs,av}(f < 3\,GHz)$$

In order to estimate the effect of a fraction (*p* ∈ [0, 1]) of the RF-EMF incident fields shifting to frequencies higher than 3 GHz we also determine the average *P*_*abs*_ for frequencies higher than 3 GHz, using:

6 $${P}_{abs,av}(f > 3\,GHz)=\frac{1}{5}\,\mathop{\sum }\limits_{j=1}^{5}\,{P}_{abs}({f}_{j})$$

with *f*_*j*_ = 6, 12, 24, 60,120 *GHz*. The realistic *P*_*abs*,*real*_(*p*) for a fraction *p* of the power shifted to frequencies higher than 3 GHz is then calculated as:

7 $$\begin{array}{rcl}{P}_{abs,real}(p) & = & p\times \frac{{E}_{RMS,avg}^{2}}{1\,{V}^{2}/{m}^{2}}\times {P}_{abs,av}(f > 3\,GHz)+(1-p)\\  &  & \times \,\frac{{E}_{RMS,avg}^{2}}{1\,{V}^{2}/{m}^{2}}\times {P}_{abs,av}(f < 3\,GHz)\end{array}$$

## Results

### Numerical simulations

**Figure 4:**
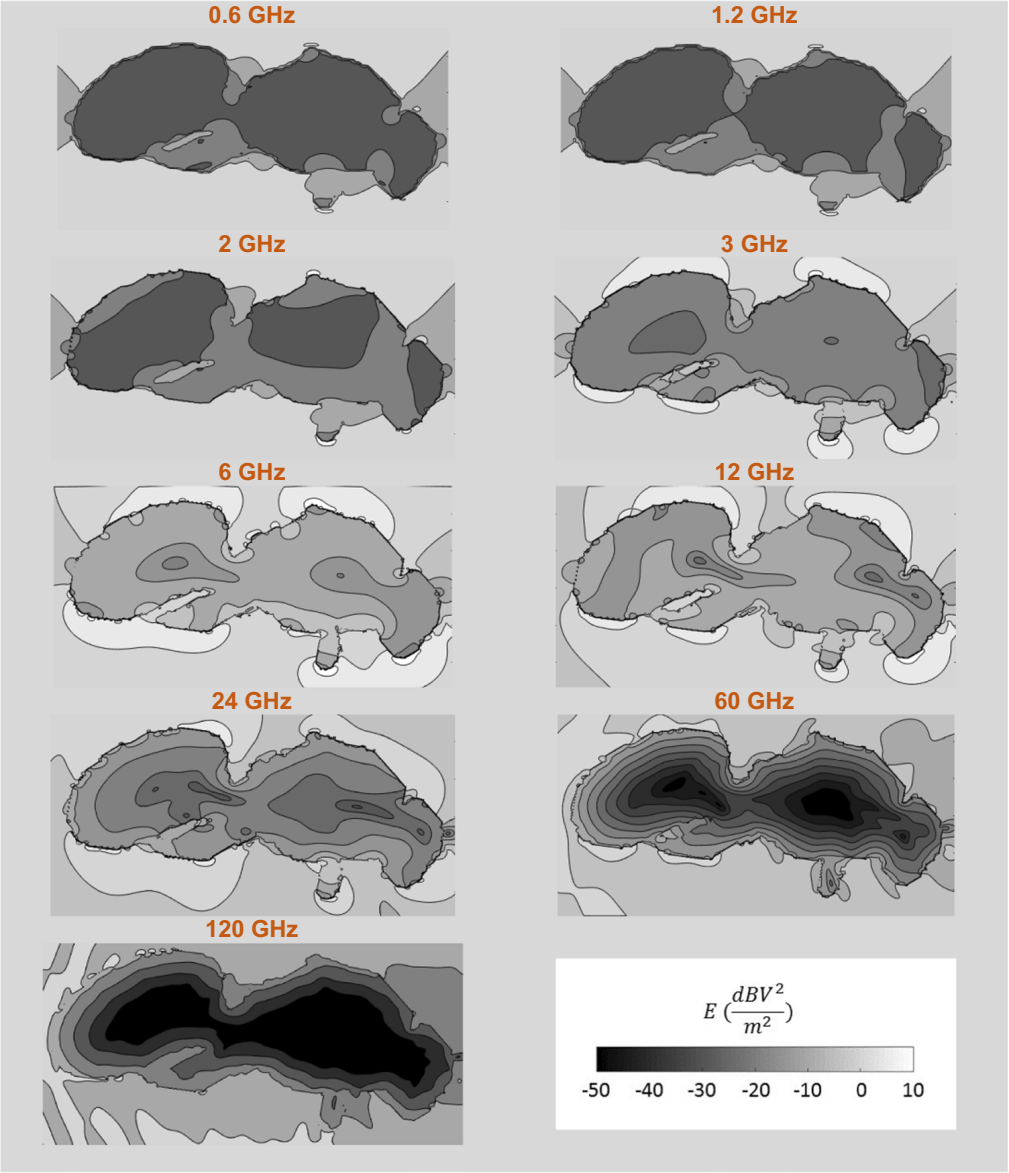
Relative electric field strength in and around a mid-sagittal plane of the Honey Bee Drone at the nine studied frequencies. Grey scale shows the electric field strengths relative to 1 V/m electric field strength.

Figure 4 shows the relative electric field strength (electric field strength divided by the maximum electric field strength in the simulation domain) in and around the studied drone in a mid-sagital plane as function of frequency for exposure configuration number 1 shown in Fig. 2. The internal electric fields increase up to 12 GHz and shift towards the outside of the phantom at higher frequencies. At 120 GHz the electric field strengths decreases very rapidly within the phantom and electric fields are basically only present in the outer layers of the insect. This is caused by a decrease in skin depth that is driven by the increase in conductivity at higher frequencies, see Table 1. Note that the total RF-EMF absorbed power in the insect scales both with the internal electric field strength and the conductivity.

**Figure 5:**
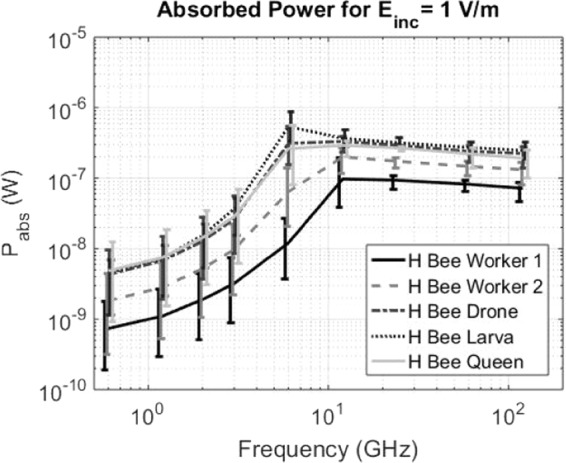
Total absorbed power (*P*_*abs*_) in the five studied honey bees as function of frequency, normalized to an incident plane-wave field strength of 1 V/m at each frequency. The curves indicate the mean values over the twelve plane wave simulations, while the whiskers indicate the maximum and minimum values found at each frequency. The whiskers are slightly offset in order to avoid visual overlap but are all determined at the simulated frequencies described in the Methods Section.

Figure 5 shows the normalized RF-EMF *P*_*abs*_ as a function of frequency for the five studied insects from 0.6 GHz up to 120 GHz. The curves connect the linear averages of the 12 *P*_*abs*_ values obtained for each honey bee at each simulated frequency, while the whiskers indicate the minimum and maximum *P*_*abs*_ values found at those frequencies. All *P*_*abs*_ values are normalized to an incident field strength of 1 V/m. Figure 5 shows an increase of *P*_*abs*_ over frequency for all studied phantoms up to 6 GHz. When comparing the average *P*_*abs*_ at 0.6 GHz and 6 GHz, we found relative increases of factors of 16, 35, 72, 121, and 54 for the Worker Bee 1, Worker Bee 2, Drone, Larva, and queen Bee, respectively. The *P*_*abs*_ slightly decreases over frequency beyond 12 GHz for all the studied honey bees. When comparing *P*_*abs*_ at 12 GHz and 120 GHz, we found relative decreases of 26%, 34%, 33%, 32%, and 34% for the Worker Bee 1, Worker Bee 2, Drone, Larva, and Queen Bee, respectively. The spread on the *P*_*abs*_ values obtained at each individual frequency reduces from up to a factor of 13 below 12 GHz to smaller than a factor 2.5 beyond 12 GHz. Figure 5 shows a general increase of *P*_*abs*_ with increasing volume and surface area of the studied insects. Previous studies on whole-body averaged absorbed RF power and specific absorption rate of humans have shown a dependency of these quantities on the absorption cross section, a quantity that scales with volume and/or surface area of an exposed subject. When the diagonals of the smallest rectangular brick that contain the insect phantoms are considered, the honey bee with the smallest diagonal, Worker Bee 1 with a diagonal of 13 mm has the overall lowest average *P*_*abs*_. The Larva, Queen Bee, and Drone all have associated diagonals of 22 mm and have similar average *P*_*abs*_ values as function of frequency. The Worker Bee 2 has a diagonal that falls in between Worker 1 and the other insects of 16 mm and also has an average *P*_*abs*_ that falls in between the curve for the smaller worker and the other honey bee models, see Fig. 5. We attribute he differences between the two Worker Bee phantoms mainly to the difference in size of both phantoms. The larger Worker Bee 2 phantom has a larger diagonal, surface area, and volume. This leads to a higher absorption cross section^33^ and higher *P*_*abs*_.

The maximal *P*_*abs*_ for the five studied insect models occurs at those wavelengths that are close to the double of this diagonal, which suggests an absorption peak around half a wavelength. The maximum *P*_*abs*_ for the Larva model lies in between 3 and 12 GHz, i.e. in between 25 and 100 mm in terms of *λ*, while the diagonal of said bounding box is 22 mm for the phantom. For the other studied insect models the maximum *P*_*abs*_ lies in between 6 and 24 GHz, i.e. in between 23 and 50 mm in terms of *λ*, with associated phantom diagonals ranging from 16 mm to 22 mm.

As mentioned in the Methods section, the influence of dielectric parameters was studied with simulations using Worker 2 at 2 GHz with altered dielectric parameters. These resulted in *P*_*abs*_ values of 6.3 × 10^−10^ W, 6.3 × 10^−9^ W, 3.1 × 10^−9^ W, and 1.8 × 10^−9^ W, in comparison to 2.0 × 10^−9^ W for an incident field strength of 1 *V*/*m*. This corresponds to relative deviations of −69%, +210%, +50%, and −10%. These deviations are significant but smaller than the full range of a factor of 5 we observed for the larva at 2 GHz as a function of changing incident angle and polarization. These relative differences are small in comparison to the differences we observe over frequency for the same phantom: a factor of 121 over frequency from 0.6 to 6 GHz.

At 120 GHz we find a deviation on *P*_*abs*_ smaller than 0.1% when 120 simulation periods are executed in comparison to 30 simulation periods in configuration number 2 shown in Fig. 2 for the Worker 2 phantom. Indicating that the number of simulated periods is sufficient for these simulations. At the same frequency and in the same simulation configuration, a reduction of the grid step with a factor of 2 resulted in a *P*_*abs*_ of 8.6 × 10^−8^ W and 3.1 × 10^−7^ W for the Worker 2 and Larva phantoms, respectively, while the regular simulations with 0.1 mm and 0.05 mm grid steps, respectively, resulted in *P*_*abs*_ values of 8.4 × 10^−8^ W and 3.1 × 10^−7^ W for an incident field strength of 1 *V*/*m*. This corresponds to relative deviations of 0.3% and 0.5% for the Worker 2 and the Larva phantoms, respectively, indicating that the chosen grid step was small enough to result in stable numerical results.

The set of 20 incident plane waves with randomized angles of incidence and polarization at 6 GHz using the Worker 2 phantom resulted in an average *P*_*abs*_ of 4.5 × 10^−8^ ± 1.6 × 10^−8^ W for an incident field strength of 1 *V*/*m*, while the set of 12 incident plane waves used to model far-field exposure results in an average *P*_*abs*_ of 6.5 × 10^−8^ ± 5.3 × 10^−8^ W at the same frequency. The value are fairly close, which indicates that the set of 12 incident plane waves along the main axes is a good proxy for average exposure under a randomized angle of incidence and polarization. The set of twelve plane waves does seem to overestimate exposure at the higher percentiles, since they are significantly higher than those obtained using the random set of plane waves.

### RF-EMF field measurements

**Figure 6:**
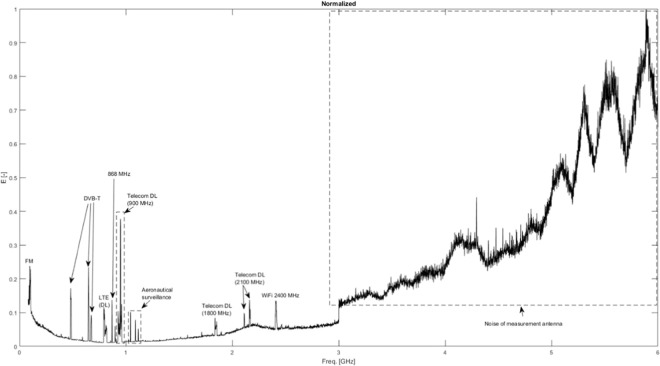
Overview measurement of electric field strength (normalized to maximally measured electric field strength), between 0.8 and 6 GHz, in Aalter. The wireless technologies associated with the different peaks are indicated in the figure as well.

Figure 6 shows an example of an RF-EMF overview measurement at one of the five studied bee hives (Aalter). Figure 6 shows the relative electric field strength, normalized to the maximally measured electric field strength. The different peaks correspond to several individual frequency bands that are used for telecommunication and broadcasting signals. These frequency bands were then measured individually using the same set-up with triaxial antenna and spectrum analyzer at two positions relative to the bee hive on each measurement site using the measurement procedure described in^32^.

Table 2 lists the measured *E*_*RMS*_ values at the five studied bee hives shown in Fig. 3. As all these measurement sites were rural, private areas, there were no uplink (emissions from a user device to the network) transmissions found. Downlink (DL, this is network to user communication) signals were found at all measurement sites. These signals were generated by three different mobile telecommunications providers in fourteen different frequency bands. The wireless technologies used by the telecommunication operators were: Long Term Evolution (LTE) in frequency bands close to 800 MHz and 1800 MHz, Global System for Mobile telecommunications (GSM) in frequency bands close to 900 MHz, and Universal Mobile Telecommunications Service (UMTS) in frequency bands close to 900 MHz and 2100 MHz. Four other telecommunication bands were identified: TETRA (Terrestrial Trunked Radio, 390–395 MHz) which is a technology used by public services (police, firefighters, etc.), an Industrial, Scientifical, and/or Medical (ISM) application around 870 MHz, Digital Enhanced Cordless Telecommunications (DECT) close to 1900 MHz, and Wireless Fidelity (WiFi) at 2400 MHz. Additionally, several frequency bands with RF signals for broadcasting were measured: Frequency Modulated (FM) Radio around 100 MHz, Digital Audio Broadcasting (DAB) around 200 MHz, Digital Video Broadcasting (DVB) at 480–680 MHz. We found one unidentified RF wireless transmission at 592 MHz on two measurement sites: Merelbeke and Eeklo. The total *E*_*RMS*_ values ranged from 0.016 V/m on both positions in Merelbeke up to 0.226 V/m on position 1 in Drongen. The average *E*_*RMS*_ over the ten studied measurement sites was 0.06 V/m. FM Radio was the dominant source of RF exposure on 7/10 measurement positions. In Drongen and in Aalter, GSM 900 DL was the dominant contributor to the RF-EMF exposure. The field strength of WiFi signals depends strongly on the duty cycle used by the wireless technology^34^. The measured *E*_*RMS*_ values can be extrapolated to peak values under the assumption of 100% duty cycle. In the case of Aalter, this would result in 0.027 V/m and 0.032 V/m on positions 1 and 2, respectively. In the case of Zomergem, this extrapolation would result in peak *E*_*RMS*_ values of 0.059 V/m and 0.016 V/m on positions 1 and 2, respectively. On both measurement sites, a theoretically maximal 90% duty cycle would make WiFi the dominant source of exposure. However, such a network load is unlikely in a rural area. WiFi was not measured at three out of five measurement sites. Additionally, at all measurement sites, RF EMFs emitted by a pulsed radar or other wireless technologies used in aeronautical surveillance were observed. The *E*_*RMS*_ value of RF EMFs emitted by a radar cannot be accurately measured without having the specifications of the radar. Therefore, we can only measure the peak value over the 6 min measurement interval. These fields were the highest in Merelbeke, where at position 1 peak E-field values of 0.017 V/m and 2.2 V/m were measured at 1.09 GHz and 1.3 GHz, respectively, while at position 2 peak E-field values of 0.02 V/m and 2.9 V/m were measured at at 1.09 GHz and 1.3 GHz, respectively.

**Table 2:** Measured root-mean squared electric field strengths (*E*_*RMS*_) in the $80\,MHz-6\,GHz$ frequency band in V/m.

*E*_*RMS*_(*V*/*m*)	Aalter		Merelbeke		Eeklo		Zomergem		Drongen	
Frequency Band	Pos 1	Pos 2	Pos 1	Pos 2	Pos 1	Pos 2	Pos 1	Pos 2	Pos 1	Pos 2
FM^*a*^ radio	**0.019**	**0.021**	**0.009**	**0.009**	**0.018**	**0.014**	**0.011**	**0.011**	0.009	0.008
T-DAB	—^*b*^	—	—	—	—	—	0.004	0.005	0.005	0.004
TETRA (390 MHz- 395 MHz)	0.001	0.001	0.002	0.001	0.001	0.001	—	—	0.001	0.002
DVB-T 482 MHz	0.009	0.006	—	—	0.003	0.003	0.008	0.006	0.004	0.002
Freq. 592 MHz	—	—	0.001	0.002	0.002	0.002	—	—	—	—
DVB-T 650 MHz	0.008	0.008	0.003	0.003	0.002	0.003	0.006	0.006	0.006	0.004
DVB-T 674 MHz	0.004	0.008	0.004	0.004	0.002	0.002	0.006	0.005	0.004	0.004
ISM 868 MHz (869.5 MHz)	0.001	0.001	—	—	—	—	—	—	—	—
LTE 800 DL Prov. 1^*c*^	0.003	0.004	0.001	0.001	0.006	0.004	0.002	0.002	0.002	0.002
LTE 800 DL Prov. 2	0.002	0.002	0.004	0.004	0.002	0.002	0.002	0.002	0.047	0.031
LTE 800 DL Prov. 3	0.003	0.002	0.001	0.001	0.002	0.002	0.002	0.002	0.087	0.073
GSM 900 DL Prov. 1	0.005	0.004	0.001	0.002	0.005	0.007	0.003	0.004	0.004	0.004
GSM 900 DL Prov. 2	**0.019**	**0.036**	0.008	0.009	0.002	0.003	0.003	0.004	0.065	0.083
GSM 900 DL Prov. 3	0.004	0.004	0.003	0.002	0.002	0.003	0.003	0.004	**0.180**	**0.137**
UMTS 900 DL Prov. 1	0.001	0.002	0.001	0.001	0.003	0.003	0.002	0.002	0.002	0.001
UMTS 900 DL Prov. 2	0.001	0.001	0.005	0.006	0.001	0.001	0.001	0.001	—	—
UMTS 900 DL Prov. 3	0.002	0.002	0.001	0.001	0.001	0.001	0.002	0.001	0.055	0.055
LTE 1800 DL Prov. 1	—	—	—	—	0.004	0.005	—	—	—	—
LTE 1800 DL Prov. 3	0.004	0.004	—	—	—	—	—	—	—	—
DECT 1880 MHz	—	—	—	—	—	—	0.002	0.003	0.002	0.001
UMTS 2100 Prov. 1	—	—	—	—	0.006	0.007	—	—	—	—
UMTS 2100 DL Prov. 2	0.003	0.003	0.004	0.004	—	—	—	—	0.039	0.026
UMTS 2100 Prov. 3	0.005	0.006	—	—	—	—	—	—	—	—
WiFi 2400 MHz instantaneous^*d*^	0.007^*e*^	0.008^*e*^	—	—	—	—	0.006^*f*^	0.002^*f*^	—	—
**Total instantaneous**	**0.032**	**0.046**	**0.016**	**0.016**	**0.022**	**0.020**	**0.019**	**0.018**	**0.226**	**0.189**

^*a*^‘FM’ = Frequency Modulated,’TETRA’ = Terrestrial Trunked Radio, ‘DVB-T’ = Digital Video Broadcasting - Terrestrial, ‘ISM’ = Industrial, Scientifical, and Medical’LTE’ = Long Term Evolution, ‘GSM’ = Global System for Mobile Communication, ‘UMTS’ = Universal Mobile Telecommunications System, ‘DECT’ = Digital Enhanced Cordless Telecommunications, ‘WiFi’ = Wireless Fidelity.

^*b*^‘—’ indicates that the frequency band was not present at the measurement site.

^*c*^Three identified Providers are denoted as Prov. 1, 2, and 3.

^*d*^*E*_*RMS*_ values for Wireless Fidelity (WiFi)depend on the used duty-cycle, which depends on the use of the network.

^*e*^Duty cycle of 7%.

^*f*^Duty cycle of 1%.

In order to provide the readers with a complete overview of the exposure to EMF fields below 6 GHz at the chosen measurement sites, Table 3 lists measured values in the 100 kHz to 6 GHz range using a broadband field probe. All the average values are higher than what is obtained from the frequency-selective measurements presented in Table 2, as should be the case since a broader band is considered.

**Table 3:** Measured maximum and time-averaged broadband incident electric field strengths ($100\,kHz-6\,GHz$).

Location	Maximum E-field (1 s interval) (V/m)	Avg E-field (1 s interval) (V/m)
Aalter	0.430	0.272
Merelbeke	0.233	0.1675
Eeklo	0.652	0.532
Zomergem	0.665	0.346
Drongen	0.397	0.297
Average	0.503	0.344

### Estimation of realistic RF-EMF absorbed power in honey bees

Using the results presented in Table 2, one can rescale the *P*_*abs*_ values shown in Fig. 5 in order to obtain a realistic estimate of the absorbed RF-EMF power in honey bees *P*_*abs,real*_. The third to eight columns of the top row of Table 4 list *P*_*abs,real*_ assuming that all incident *E*_*rms*_ = 0.06 *V*/*m* is uniformly distributed over the simulated *P*_*abs*_ values lower than 3 GHz. These values range from 0.1 nW for Worker 1 until 0.7 nW for the Larva and Queen Bee. In each subsequent row, 10% of the incident power density is transferred to frequencies higher than 3 GHz. This causes an increase in the estimated *P*_*abs,real*_(*p*). In order to quantify this increase, the five columns to the right show the relative increase in *P*_*abs,real*_(*p*) as *p* increases from 0 to 1. A full shift of all RF-EMF power to frequencies higher than 3 GHz - without changing the incident field strength - would result in relative increases in absorbed power between a factors 24–48 for the studied honey bee models. Even a relatively small shift of 10% of the incident power density to higher frequencies will lead to a relative increase in *P*_*abs*_ of a factor higher than 3, see Table 4.

**Table 4:** Absorbed power in the four studied insects for an incident electric field strength of 0.06 V/m, distributed uniformly over frequencies lower and higher than 3 GHz for different relative fractions.

Fraction < 3 *GHz* (1 − *p*) (%)	Fraction > 3 *GHz p*(%)	*P*_*abs*,*real*_(*p*)(*nW*)					$\tfrac{{{\boldsymbol{P}}}_{{\boldsymbol{abs}},{\boldsymbol{real}}}({\boldsymbol{p}})}{{{\boldsymbol{P}}}_{{\boldsymbol{abs}},{\boldsymbol{real}}}(100{\boldsymbol{ \% }} < {\bf{3}}\,{\boldsymbol{GHz}})}(.\,)$				
		Drone	Worker 1	Worker 2	Larva	Queen Bee	Drone	Worker 1	Worker 2	Larva	Queen Bee
100	0	0.63	0.010	0.26	0.73	0.71	1	1	1	1	1
90	10	2.5	0.57	1.2	3.0	2.3	3.9	5.7	4.6	4.2	3.3
80	20	4.3	1.0	2.1	5.3	3.9	6.8	10	8.2	7.4	5.6
70	30	6.2	1.5	3.1	7.6	5.6	9.7	15	12	11	7.8
60	40	8.0	2.0	4.0	9.9	7.2	13	20	15	14	10
50	50	9.8	2.4	5.0	12	8.8	16	25	19	17	12
40	60	12	2.9	5.9	15	10	18	29	23	20	15
30	70	14	3.4	6.9	17	12	21	34	26	23	17
20	80	15	3.9	7.8	19	14	24	39	30	26	19
10	90	17	4.3	8.8	22	15	27	43	33	30	21
0	100	19	4.8	9.7	24	17	30	48	37	33	24

## Discussion

This study investigates RF-EMF absorption in Western Honey Bees as a function of frequency in the 0.6 to 120 GHz range. To this aim, we used five different models of different honey bees: two workers, a drone, a larva, and a queen. These models were obtained using micro-CT imaging and used for FDTD simulations. These were used to evaluate far-field exposure of honey bees. This far-field exposure is modeled as a set of plane waves at harmonic frequencies between 0.6 and 120 GHz. The numerical simulations resulted in *P*_*abs*_ as a function of frequency for the different studied honey bees. These simulations were combined with real RF-EMF exposure measurements near bee hives in Belgium in order to estimate realistic exposure values for honey bees.

Micro-CT imaging is a technique that has previously been shown to accurately scan insects^35,36^. The models used in this study have resolutions between 0.02 *mm* and 0.25 *mm*, which is larger than the resolution of the micro-CT models using in^11^. Since the smallest grid step used in our simulations is 0.05 *mm*, the ideal resolution of the insect models would be smaller than that. The larger resolution of the scanning is not a problem for the stability of the FDTD algorithm, but more spatial resolution could be obtained with the same simulation settings. It is expected that the micro-CT models used in this study lead to a better estimation of *P*_*abs*_ and the spatial distribution of the electric fields than approximate models such as ellipsoids or cylinders^37^.

The results of our numerical simulations, see Fig. 5, show an increase of *P*_*abs*_ with frequency up to 6–12 GHz. Figure 4 illustrates the mechanism behind this increase: as the frequency increases the EMFs are less likely to diffract around the honey bees, that are relatively small in comparison to the wavelengths <6 *GHz*, and can penetrate further in the models, generating higher internal electric fields and consequently higher *P*_*abs*_ values. Figure 4 also shows why the whole-body averaged *P*_*abs*_ does not increase beyond 12 GHz. As the conductivity increases, see Table 1, the electric fields will decay faster within the honey-bee phantoms, which leads to larger relative volumes within the insect with lower fields, see Fig. 4, which will also contribute to the whole-body averaged *P*_*abs*_. This effect also causes the *P*_*abs*_ to have a smaller dependency (variation) on incident angle and polarization, see Fig. 5. We also observe that both the frequency-dependency of the *P*_*abs*_, i.e. the transition point between sharp increase in *P*_*abs*_ over frequency and slight decrease over frequency, and the magnitude of the *P*_*abs*_, i.e. the offset of the *P*_*abs*_ curve, depend on the honey bee’s size. This effect was previously observed in^11^. In general, the results presented in this manuscript are in excellent agreement with those presented in^11^. The results in terms of *P*_*abs*_ obtained for the honey bees in this study fall right in between those obtained in^11^ for the smaller Australian Stingless Bee and the larger Desert Locust, which confirms again the dependency of *P*_*abs*_ on phantom size. The same size-related effect was described for humans in^28,33,38^ and comparable frequency trends were observed in humans that have larger full-body sizes at MHz frequencies^28,38^. It should be noted that this manuscript focused on exposure of individual insects in free space. In reality, honey bees might cluster, creating a larger absorption cross section and potentially higher absorption at lower frequencies.

The FDTD simulations presented in this manuscript use dielectric properties that were obtained from the literature survey executed in^11^. Ideally, these dielectric parameters would be obtained for the honey bees studied in this manuscript. However, as shown in^11^, most studies on dielectric properties of insects in literature^3,39–41^ show similar frequency dependencies of those dielectric parameters. We have executed additional numerical simulations to test for the uncertainty on the dielectric parameters and found deviations up to 210% on *P*_*abs*_, which is significant but still smaller than the variations that exist due to changing angle of incidence and polarization at a fixed frequency, or changes in frequency. We modeled the insects as homogeneous dielectric objects, while in reality they have heterogeneous dielectric parameters. Even though the FDTD algorithm will always require an averaging of dielectric parameters over the cube size, further developments in honey bee and insect phantoms should be focused on the inclusion of multiple tissues in order to refine these models.

*In-situ* RF-EMF measurements were executed using a measurement set up consisting out of a spectrum analyzer connected to an isotropic, triaxial antenna according to the measurement procedure listed in^32^. We measured total incident *E*_*RMS*_ between 0.016 V/m and 0.226 V/m in five rural environments with a linear average of 0.06 V/m and a quadratic average of 0.1 V/m. Joseph *et al*.^32^ measured a median total *E*_*RMS*_ value of 0.09 V/m over several rural locations in Belgium, the Netherlands, and Sweden. Bhatt *et al*.^1^ measured an average *E*_*RMS*_ value of 0.07 ± 0.04 *V*/*m* in rural environments in Belgium. Both previous studies of rural RF-EMF exposure are close to what we found in this manuscript and certainly within the measurement uncertainty of 3 dB on our measurements.

As our RF-EMF exposure measurements near bee hives demonstrate, see Table 2, most of the current RF-EMF exposure is located at frequencies ≤1 *GHz*. Additionally, Fig. 5 demonstrates that the *P*_*abs*_ in all studied Honey bee models is lowest at frequencies ≤1 *GHz*. This implies that in reality, potential shifts in telecommunication frequencies to higher frequencies might induce even larger increases that the ones estimated in Table 4 since in that analysis an average value over all *P*_*abs*_ values ≤3 *GHz* is assumed.

### Strengths and limitations

This manuscript presents several contributions to the state of the art in the field of RF-EMF exposure assessment of insects. First, to the best of the authors’ knowledge, this is the only paper where a numerical RF dosimetry is presented for different developmental stages of honey bees. Second, this is the only study that combined real, *in-situ* exposure measurements with numerical simulations of RF-EMF exposure of insects in order to estimate a realistic exposure of honey bees. In comparison to our previous study^11^, we considered a broader frequency range from 0.6 GHz up to 120 GHz, which is more in line with the frequencies used in the current telecommunication networks (3 G and 4 G). Finally, this study presents a unique quantification of real-life exposure of honey bees and estimations of how this might change if future frequency shifts in that exposure might occur. A disadvantage of this study is that we did not executed dielectric and thermal measurements in order to obtain dielectric and thermal properties of the studied honey bees. We obtained dielectric properties from literature and were able to execute electromagnetic simulations. We did not perform thermal simulations in this study. Another disadvantage is that we modeled far-field exposure by a limited number of plane waves, while previous studies have shown that a large set of plane waves is necessary to properly model far-field exposure^26^. We did executed a validation of our exposure set up by comparing it with a set of random plane wave exposures and found good correspondence, certainly close to the mean/median. Finally, we used FDTD simulations that are faced with uncertainties^29^ and used models that have a limited spatial resolution. This is a disadvantage of any RF-EMF simulation study in comparison to a study that relies on measurements of real insects.

### Future research

Our future research will focus on executing exposure measurements of insects in order to validate the RF-EMF *P*_*abs*_ values and the dielectric parameters. Additionally, we would like to execute thermal simulations of honey bees and other insects under RF-EMF exposure. Finally, we aim to work on the development of more insect phantoms, with more spatial accuracy and potentially several independently identified tissues.

## Conclusions

Exposure of Western Honey Bees (*apis mellifera*) to radio-frequency (RF) electromagnetic fields was studied using a combination of *in-situ* exposure measurements near bee hives in Belgium and numerical simulations. The simulations use the finite-difference time-domain technique to determine the electromagnetic fields in and around five honey bee models exposed to plane waves at frequencies from 0.6 GHz up to 120 GHz. These simulations lead to a quantification of the whole-body averaged absorbed radio-frequency power (*P*_*abs*_) as a function of frequency. The average *P*_*abs*_ increases by factors 16 to 121, depending on the considered phantom, when the frequency is increased from 0.6 GHz to 6 GHz for a fixed incident electric field strength. A relatively small decrease in *P*_*abs*_ is observed for all studied honey bees between 12 and 120 GHz. RF exposure measurements were executed on ten sites near five different locations with bee hives in Belgium. These measurements resulted in an average total incident RF field strength of 0.06 V/m, which was in excellent agreement with literature. This value was used to assess *P*_*abs*_ for those honey bees at those measurement sites. A realistic *P*_*abs*_ is estimated to be between 0.1 and 0.7 nW for the studied honey bee models. Assuming that 10% of the incident power density would shift to frequencies higher than 3 GHz would lead to an increase of this absorption between 390–570%. Such a shift in frequencies is expected in future networks.
